# HJURP antagonizes CENP-A mislocalization driven by the H3.3 chaperones HIRA and DAXX

**DOI:** 10.1371/journal.pone.0205948

**Published:** 2018-10-26

**Authors:** Jonathan Nye, David Sturgill, Rajbir Athwal, Yamini Dalal

**Affiliations:** Chromatin Structure and Epigenetics Mechanisms Unit, Center for Cancer Research, National Cancer Institute National Institutes of Health, Bethesda, MD, United States of America; Geisel School of Medicine at Dartmouth, UNITED STATES

## Abstract

The centromere specific histone H3 variant CENP-A/CENH3 specifies where the kinetochore is formed in most eukaryotes. Despite tight regulation of CENP-A levels in normal cells, overexpression of CENP-A is a feature shared by various types of solid tumors and results in its mislocalization to non-centromeric DNA. How CENP-A is assembled ectopically and the consequences of this mislocalization remain topics of high interest. Here, we report that in human colon cancer cells, the H3.3 chaperones HIRA and DAXX promote ectopic CENP-A deposition. Moreover, the correct balance between levels of the centromeric chaperone HJURP and CENP-A is essential to preclude ectopic assembly by H3.3 chaperones. In addition, we find that ectopic localization can recruit kinetochore components, and correlates with mitotic defects and DNA damage in G1 phase. Finally, CENP-A occupancy at the 8q24 locus is also correlated with amplification and overexpression of the MYC gene within that locus. Overall, these data provide insights into the causes and consequences of histone variant mislocalization in human cancer cells.

## Introduction

The kinetochore is essential for proper chromosome segregation during mitosis. It forms the microtubule binding interface on each chromosome allowing sister chromatids to separate during anaphase. The kinetochore is formed at a defined region on each chromosome called the centromere. In most organisms besides budding yeast, which has a point centromere defined by a specific DNA sequence, this region is made up of complex repetitive DNA elements [1]. All human centromeres contain ~171 bp repeats called alpha satellite DNA. This conservation might suggest that these repetitive elements play a role in centromere identity. However, because new centromeres exist at sites that do not contain repetitive sequences, centromeres are thought to be specified epigenetically by the presence of nucleosomes containing the histone H3 variant CENP-A [2]. Indeed, it has been demonstrated that the assembly of CENP-A into chromatin is sufficient to build a functional kinetochore [3, 4].

Interestingly, CENP-A is also overexpressed in a wide variety of tumors [5–7]. In a recent analysis of 12 different types of human cancers, overexpression of CENP-A occurred 85% of the time when compared to normal tissue [8]. In addition, even modestly increased CENP-A levels are significantly correlated with increased tumor grade and poor patient outcome [9–11]. Consequently, CENP-A expression levels are now routinely included as part of a biomarker panel to determine whether breast cancer patients should undergo chemotherapy [12]. Given its potential importance to human health, it is of interest to dissect the mechanisms that permit overexpressed CENP-A to localize to ectopic sites, and to uncover potential consequences of such mislocalization in cancer cells.

Normally, CENP-A is deposited at the centromere via its association with the chaperone HJURP [13–15]. In contrast, recent work has reported that overexpression of CENP-A in HeLa cells resulted in binding of CENP-A to the H3.3 chaperone DAXX and mislocalization to genomic sites outside of the centromere [16]. In this report, CENP-A mislocalization was reported to be driven by DAXX, but not by other H3.3 variant chaperones. We previously reported that innate overexpression of CENP-A in human colorectal cancer cells leads to increased ectopic CENP-A [17], a large fraction of which localizes to DNAse I hypersensitive sites (DHS) and gene promoters; a smaller fraction of which localizes to larger domains at subtelomeric breakpoints. Interestingly, H3.3 nucleosome assembly at high turnover sites is thought to be achieved by the chaperone HIRA, not DAXX. Thus, two imperative questions arise from these findings; namely, how CENP-A is incorporated at ectopic sites; and functional consequences that may arise from CENP-A mislocalization.

In order to address these questions, we took advantage of a well-characterized colorectal cancer cell line (SW480), in which we previously documented innately increased levels of endogenous CENP-A at ~2500 defined ectopic sites [17]. Unlike parallel studies, which focused on constitutive overexpression using tagged CENP-A constructs, this system allowed us to investigate the effects of relatively modest innate overexpression (~2-fold compared to normal colon cells) of endogenous CENP-A produced from its native locus. Using this system, we find that ectopic CENP-A mislocalization is promoted by both H3.3 chaperones, HIRA and DAXX. Additionally, we observe that HJURP safeguards the genome by counteracting ectopic kinetochore formation and mitotic defects brought about by the promiscuous binding of CENP-A to H3.3 chaperones. Finally, we show that stable CENP-A occupancy at the subtelomeric fragile site 8q24, results in ectopic recruitment of kinetochore proteins, and correlates with amplification and overexpression of the MYC oncogene. These data suggest a model in which balancing the relative amounts of histone variants with their associated chaperones is critical for the maintenance of genome integrity.

## Results

### CENP-A mislocalization is promoted by the H3.3 chaperones HIRA and DAXX

In previous work, induced overexpression of CENP-A in HeLa cells revealed that the H3.3 chaperone DAXX was the primary chaperone for ectopic CENP-A assembly [16, 18]. Previously, we also reported excess CENP-A was bound to DAXX in colon cancer cells [17]. However, DAXX is thought to assemble nucleosomes predominately at telomeres, repetitive elements, and heterochromatic regions of the genome, but not at gene promoters [19–21]. This is puzzling, because a significant portion of CENP-A ectopic sites, we found, occurs at regions of high nucleosome turnover, such as DHS sites [22]. Indeed, H3.3 nucleosome assembly at these sites is usually carried out by another H3.3 chaperone HIRA [23, 24]. Therefore, we systematically examined the effects of depleting HIRA and other chaperones on ectopic CENP-A localization.

First, we investigated whether in SW480 cells, innate CENP-A overexpression was a result of abnormal cell cycle expression of the CENP-A gene, which might complicate our analysis. We examined CENP-A mRNA by qRT-PCR from cells synchronized over the cell cycle. As has been previously reported for normal human cells [25], we observed the majority of CENP-A mRNA expression occurs at G2/M (S1 Fig), supporting replication-independent incorporation.

**Fig 1:**
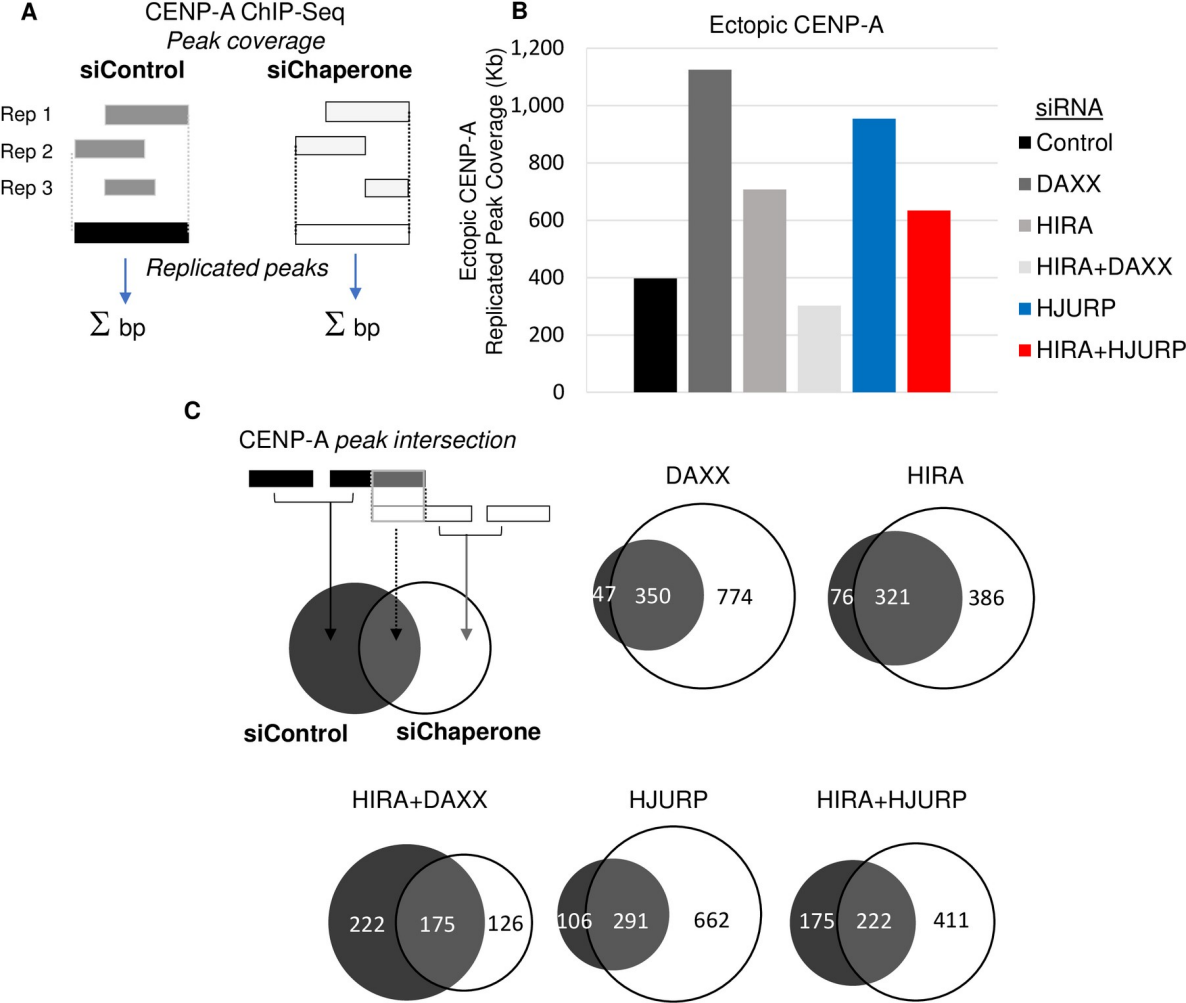
CENP-A Mislocalization requires the H3.3 chaperones HIRA and DAXX. A. Schematic of the definition of replicated peak sets and the calculation of ectopic CENP-A peak coverage. B. Bar chart showing the peak coverage, in kilobases, of replicated ectopic CENP-A peaks from 3 replicate ChIP-seq experiments after 72-hour knockdown of the indicated protein. C. Schematic of the intersection of peaks from control and chaperone knockdown samples. Proportional Venn diagrams showing the intersection between chaperone knockdown and control in kilobases of peak coverage. In each case, peak coverage that is newly acquired in the chaperone knockdown is in white.

We next performed siRNA knockdowns of the CENP-A specific chaperone HJURP, as well as two H3.3 chaperones HIRA and DAXX. After a 72-hour knockdown, we first confirmed the depletion of these proteins by Western blots (Figures A and B in S2 Fig). We next performed native CENP-A ChIP-Seq from these individual knockdowns alongside control cells, to examine potential changes in ectopic CENP-A occupancy. For this analysis, we used two methods to examine the amount of ectopic CENP-A genome wide. One method selected only the most replicated peaks from multiple replicates (Fig 1A and 1B); the other normalized for differences in read depth between replicates by pooling peak calls and using random sampling with the same number of reads (Figure A in S3 Fig). Importantly, both methods yielded similar results. In contrast to previous findings, when DAXX was depleted, we observed an increase in ectopic CENP-A (Fig 1B and Figure A in S3 Fig) [16]. Indeed, ectopic CENP-A peak coverage *increased* to 1,124 kilobases (kb), compared to 397 kb in the control siRNA sample (Fig 1B). We next performed coordinate intersections between peak sets for each sample, defining peak coverage that is “common” with control siRNA, and that which is “new” following chaperone knockdown, and found that the majority of this peak coverage was newly acquired upon DAXX depletion (Fig 1C).

Recent work has suggested a compensatory gap-filling mechanism for H3.3 deposition, wherein the two H3.3 chaperones, DAXX and HIRA, can play a semi-redundant role [26]. We were curious whether this phenomenon might extend to the context of ectopic CENP-A. To test this idea, we next performed a knockdown of HIRA; this depletion also increased ectopic CENP-A peak coverage to 708 kb relative to control siRNA. Since the knockdowns of DAXX or HIRA alone did not decrease ectopic CENP-A, we tested the possibility that both of these chaperones might promote mislocalization by being partially redundant.

Consequently, we next performed siRNA against both, HIRA and DAXX. The double knockdown of both chaperones resulted in a reduction in CENP-A peak coverage, with 302 kb represented in the double knockdown compared to 397 kb in the control (Fig 1B). In addition, depletion of both chaperones led to a significant reduction in new CENP-A peak coverage compared to the single knockdowns of HIRA and DAXX alone, dropping from 386 Kb and 774 Kb respectively, to 126 Kb (Fig 1C). Moreover, much of the peak base coverage present in the control treated cells were not present in the double knockdown, with only 175 Kb in common between the two. In contrast, both the single knockdowns of DAXX and HIRA had much more in common with control, 321 Kb and 350 Kb respectively (Fig 1C). These data suggest that innate overexpression of native CENP-A results in mislocalization that is promoted by both H3.3 chaperones, HIRA and DAXX.

A logical extension of this idea is that simply titrating CENP-A, by increasing its availability, might increase ectopic localization. Therefore, we were curious to test whether knockdown of the centromeric chaperone HJURP had an effect on ectopic CENP-A levels. Interestingly, when we depleted HJURP, this resulted in ectopic CENP-A peak coverage more than doubling, increasing from 397 kb to 954 kb in the HJURP knockdown (Fig 1B). Similar to DAXX and HIRA knockdowns, the majority of this ectopic peak coverage, 662 Kb, was not present in control (Fig 1C). We reasoned the corollary of this experiment would be that when HJURP levels are low, CENP-A may be more likely to bind to H3.3 chaperones and thereby assembled ectopically. To test this idea, we performed a double knockdown of HJURP and HIRA. This resulted in a 320 kb reduction in the ectopic CENP-A peak coverage compared to the HJURP knockdown alone, dropping from 954 kb to 634 kb. This corresponded to a 251 Kb decrease in newly acquired peak coverage compared to the HJURP knockdown alone. Together these data suggest that sufficient levels of HJURP relative to CENP-A are necessary to prevent CENP-A mislocalization, which is otherwise promoted by H3.3 chaperones.

### The H3.3 chaperone HIRA binds to endogenous CENP-A

**Fig 2:**
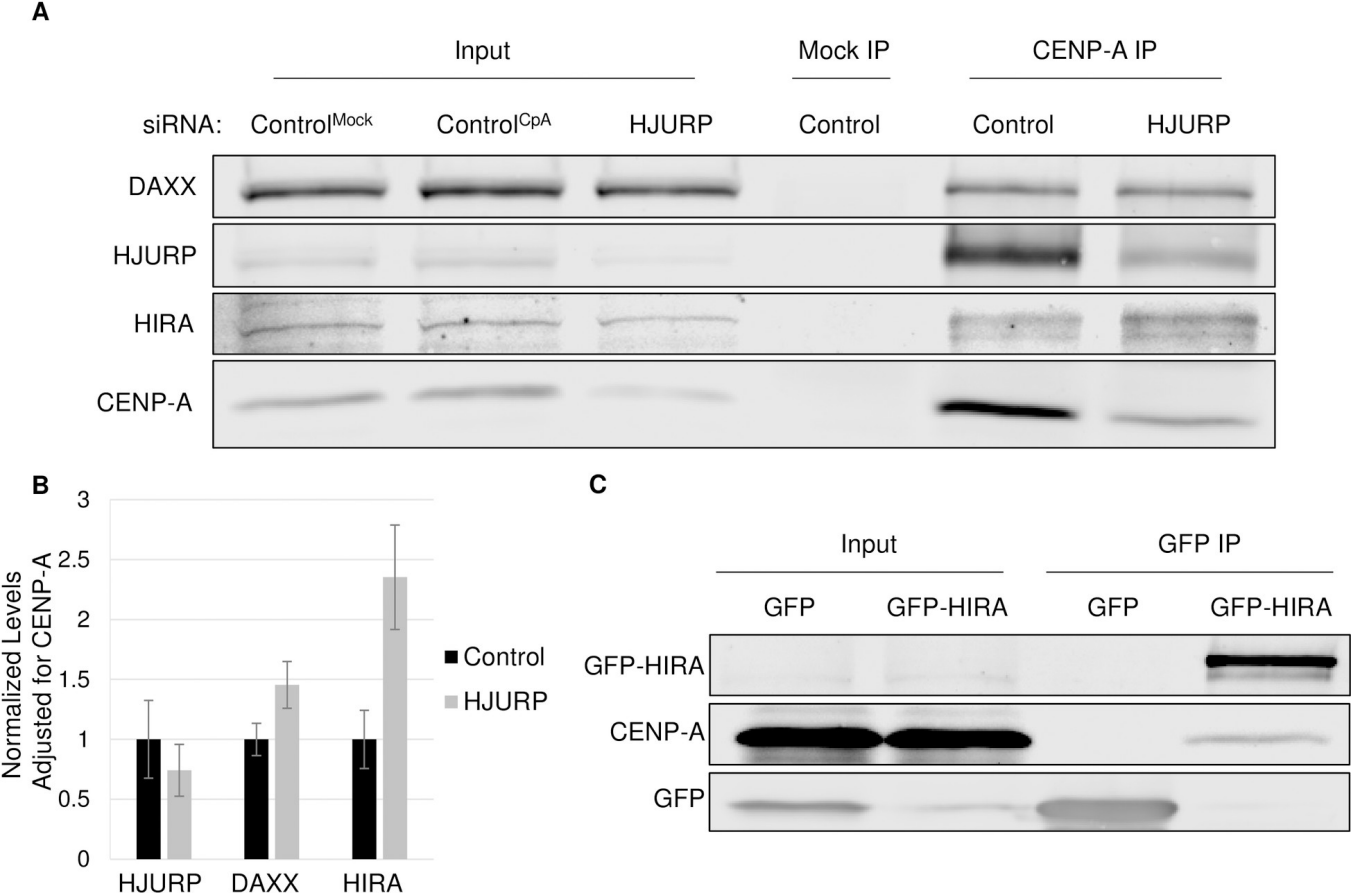
The H3.3 chaperone HIRA binds to endogenous CENP-A. A.) Western blot analysis of IPs using either CENP-A IP (ACA antibody) or Mock IP from soluble pool obtained from SW480 cells that were treated for 72 hours with either control or HJURP siRNA as indicated. Control^Mock^ IP was performed on control siRNA treated cells with no antibody while Control^CpA^ Ip was performed on control siRNA treated cells with the antibody. B.) Graph indicating the levels of each indicated chaperone in the CENP-A IP shown in A. Levels for each chaperone were normalized to control and also the amount of CENP-A pulled down in either control or HJURP siRNA experiments. Results are obtained from triplicate experiments. C.) Western blot results from IP of soluble GFP or GFP-HIRA.

One prediction that arises from these data (Fig 1) is that if the H3.3 chaperone HIRA was directly promoting CENP-A assembly at ectopic sites, it should be physically associated with soluble pre-assembly CENP-A. Concurrently, knockdown of HJURP should lead to increased binding between HIRA and CENP-A. To test this hypothesis, in cells treated with either control or HJURP siRNA, we separated the soluble fraction of CENP-A from the chromatin bound CENP-A. Using these soluble extracts, we IP’d CENP-A and probed for the presence of the H3.3 variant chaperones (Fig 2A and 2B). As expected, the centromeric chaperone HJURP co-IP’d with CENP-A robustly. In addition, both H3.3 chaperones, HIRA and DAXX also co-IP’d with CENP-A, confirming that endogenous CENP-A can bind to both. Furthermore, knockdown of HJURP resulted in a 2.4 and 1.5-fold increase in the amount of HIRA and DAXX bound to CENP-A respectively. Consistent with these findings, a reciprocal immunoprecipitation of GFP or GFP-HIRA from stable cell lines revealed that endogenous CENP-A co-IPs with HIRA, but not with GFP alone (Fig 2C). Therefore, these data suggest that both H3.3 chaperones HIRA and DAXX can bind to CENP-A, and that when HJURP is depleted, this promiscuous binding is increased.

These results differ from previous findings in which overexpressed CENP-A was shown to bind exclusively to the chaperone DAXX [16, 18]. We considered two potential explanations for this observation. First, we were curious whether HIRA may preferentially bind to endogenous CENP-A, but not to tagged versions of this protein. To test this hypothesis, we examined whether GFP-CENP-A or CENP-A-mCherry expressed in SW480 cells can bind both chaperones. Our data demonstrate that while GFP or mCherry tagged CENP-A can indeed bind to DAXX, these fusion proteins do not appear to bind HIRA (Figures A and B in S4 Fig). These data therefore suggest that HIRA binds to endogenous CENP-A, but that a tag may partially interfere with this interaction. Second, we also hypothesized that differences in chaperone abundance in different types of cancer cells may lead to a change in chaperone usage. Analysis of chaperone levels in SW480 cells revealed approximately 2.5-fold more HIRA, when compared to HeLa cells used in previous studies (Figures C-E in S4 Fig), while we only observed an approximately 1.5-fold increase in the chaperone DAXX. Therefore, importantly, these data also suggest that innate cancer-specific differences in chaperone levels may also influence CENP-A chaperone choice. A logical hypothesis arising from these data is that having the correct balance of the centromeric chaperone HJURP may play a role in suppressing ectopic CENP-A localization.

### A balance between HJURP and CENP-A is required to reduce ectopic localization

**Fig 3:**
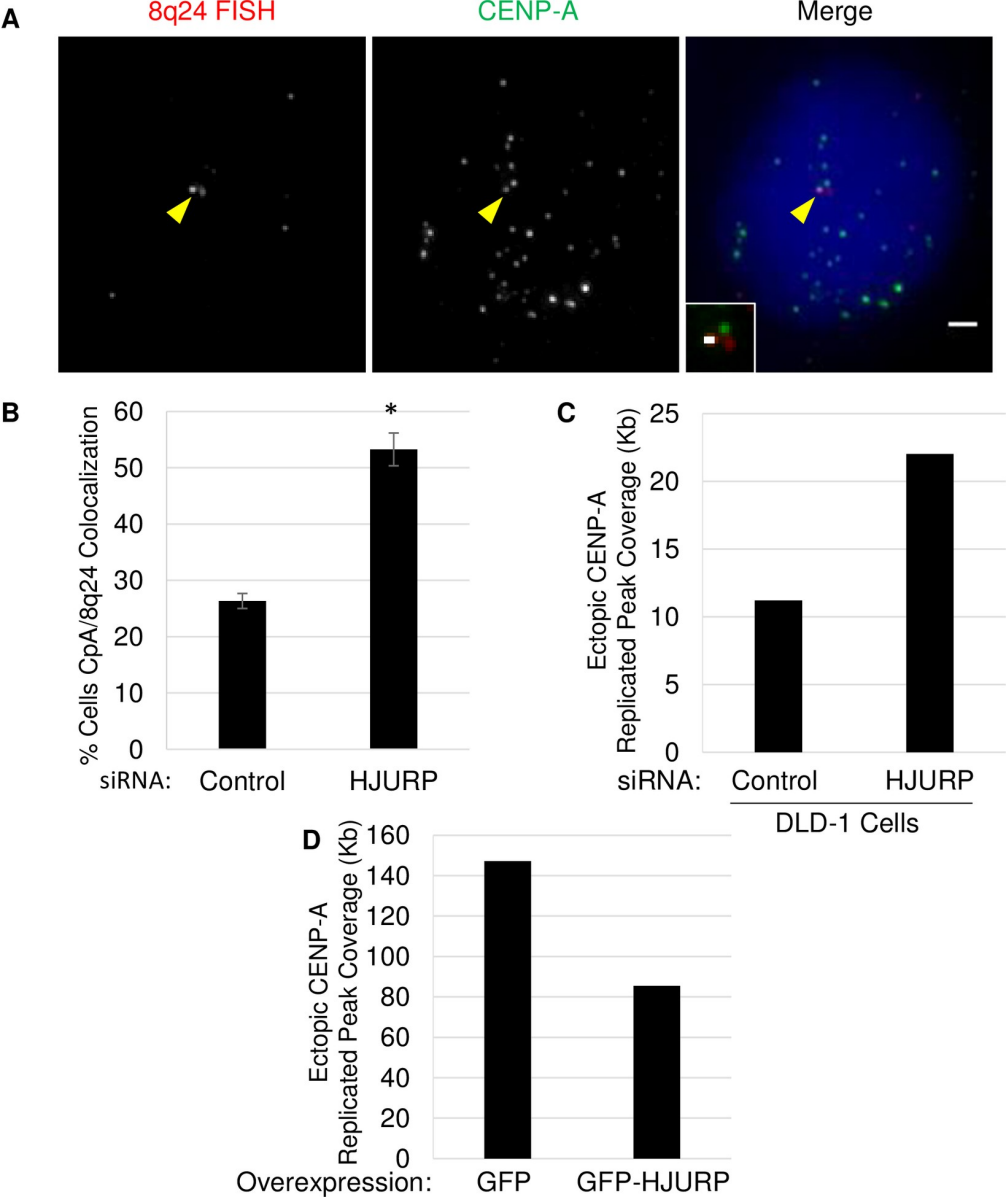
Balance of HJURP and CENP-A required to prevent ectopic localization. A.) FISH for the 8q24 locus and IF for the CENP-A protein was performed on SW480 cells. DAPI in blue. Yellow arrowheads indicate colocalization. Inset shows automated co-localization analysis performed using Image J; white is indicative of co-localization. Scale bar indicates 1μm. B.) Graph showing the results of a 72 hour knockdown of the indicated protein on colocalization of CENP-A/8q24 (*indicates P-value <0.01, Students t-test). Error bars indicate SEM from 3 experiments. N>200 cells per knockdown. C.) Graph showing the results from duplicate CENP-A ChIP-seq experiments in which cells were treated with siRNA against control or HJURP for 72-hours. Each bar represents replicated peak coverage as shown in Fig 1A. D.) Graph showing replicate peak coverage from duplicate CENP-A ChIP-seq experiments performed in stable SW480 cell lines expressing either GFP or GFP-HJURP.

Our ChIP-Seq data (Fig 1) suggested that a balance between HJURP and CENP-A must be maintained in order to reduce mislocalization. We wanted to probe this idea further. To accomplish this, we took advantage of a large ectopic CENP-A hotspot that we identified in our previous study mapping ectopic CENP-A throughout the genome [17]. This hotspot is located at the subtelomeric region 8q24, which spans the *MYC* oncogene. This region displays robust CENP-A occupancy in colon cancer cell lines in which CENP-A is naturally overexpressed (SW480, HT29), and also in early, mid, and late grade colorectal tumors, but not in normal tissues, or in cells that do not have high levels of CENP-A, such as HeLa or DLD-1 cells [17]. Ectopic CENP-A at this site can be directly visualized by combining FISH for the 8q24 locus, and IF for CENP-A (Fig 3A), yielding a simple visual read-out for CENP-A enrichment. We depleted HJURP by siRNA to test whether the loss of this chaperone influenced ectopic CENP-A at this large subtelomeric domain. After a 72-hour knockdown, we performed IF/FISH to determine the percentage of cells with CENP-A colocalization at 8q24. Consistent with previous work, we observed robust CENP-A occupancy at 8q24 in ~26.3% of control siRNA treated cells (Fig 3B). In contrast, when HJURP is depleted, we found ectopic localization of CENP-A at 8q24 doubles, from 26% to 53%. These results were consistent with the ChIP-seq results above (Fig 1), in which we observed reproducible ectopic enrichment of CENP-A in the HJURP knockdown compared to control siRNA. Moreover, our ChIP-Seq also revealed a > 4-fold increase in the number of peaks at 8q24 in the HJURP knockdown (Figures A and B in S5 Fig).

These results suggest that there needs to be a balance between CENP-A and its chaperone HJURP to prevent mislocalization. However, it was unclear whether this phenomenon occurs only in cases where CENP-A is overexpressed, or whether this phenomenon can also occur in cells with normal CENP-A expression levels, but in which HJURP is depleted. Thus, we tested this hypothesis in DLD-1 cells which we have previously shown to express normal levels of CENP-A and, consequently, exhibit very low levels of ectopic CENP-A [17]. We treated these cells with either control or HJURP siRNA for 72-hours then performed CENP-A ChIP-Seq to examine the amount of ectopic CENP-A genome wide using the same methods described above for SW480 cells (Fig 1A and Figure A in S2 Fig). This analysis revealed that replicated ectopic CENP-A peak coverage, which is already low to start with in DLD-1 cells, doubled from 11.2 Kb in the control treated cells, to 22 Kb when HJURP was depleted (Fig 3C). Indeed, this result was confirmed by our random sampling analysis as well, which showed a significant 15.6 Kb increase in peak coverage after HJURP knockdown (Figure D in S5 Fig). Thus, these data suggest that proper levels of HJURP are necessary to prevent ectopic CENP-A assembly regardless of whether this histone variant is overexpressed or not.

The logical prediction from the hypothesis above is that overexpressing HJURP should conversely reduce ectopic CENP-A. To test this idea, we generated stable cell lines that overexpress either GFP or GFP-HJURP in SW480 cells (Figures D and E in S5 Fig), and performed ChIP-seq to determine genome wide levels of ectopic CENP-A. From these experiments, we observed that HJURP overexpression reduced replicated ectopic CENP-A peak coverage from 147.2 Kb in the GFP control to 85.4 Kb (Fig 3D). Moreover, this was consistent with our random sampling analysis which showed a 74% decrease in ectopic peak coverage in the HJURP knockdown compared to control (Figure D in S5 Fig). These data provide support for the hypothesis that the correct balance between levels of CENP-A and its chaperone HJURP is essential to suppress mislocalization. In other words, when HJURP levels are limiting, soluble excess CENP-A becomes free to bind to chaperones in the H3.3 pathway.

### CENP-A mislocalization leads to recruitment of kinetochore proteins

We were next curious to explore the consequences of CENP-A mislocalization at 8q24. Recent work has shown that artificial overexpression of CENP-A can lead to chromosomal instability [18]. One proposed mechanism is that overexpression might reduce levels of kinetochore proteins at endogenous centromeres, leading to weak attachment to microtubules and mitotic defects. Interestingly, in that study, ectopic kinetochore formation was not reported [10]. This is in contrast to work in flies and fission yeast, where CENP-A overexpression results in the formation of functional ectopic kinetochores [27, 28]. Given these findings, we were curious whether in colon cancer cells, which display relatively moderate overexpression of endogenous CENP-A, and where CENP-A occupies large domains at subtelomeric sites, ectopic kinetochores can be formed.

**Fig 4:**
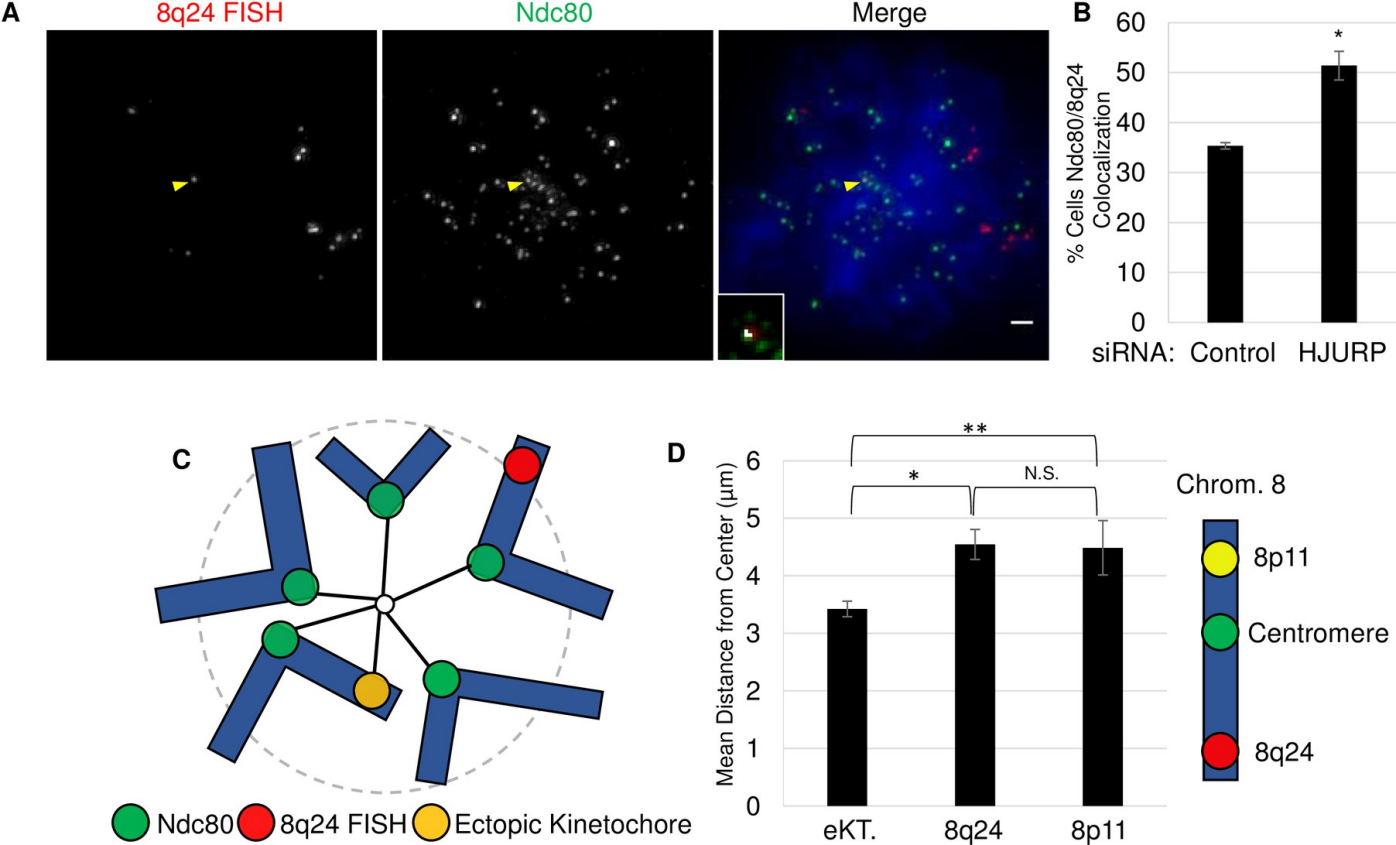
CENP-A mislocalization promotes ectopic kinetochore formation at 8q24. A.) Image showing monastrol treated cell. FISH for the 8q24 locus and IF for the Ndc80 protein was performed on SW480 cells. DAPI in blue. Yellow arrowheads indicate colocalization. Inset shows automated co-localization analysis performed using Image J; white is indicative of co-localization. Scale bar indicates 1 μm. B.) Graph showing the results of 72 hour knockdown. Percentage indicates mitotic cells with colocalization of Ndc80/8q24. Error indicates SEM from 3 experiments n>100 cells. (*indicates P-value <0.01, Students t-test). C.) Figure illustrating chromosome arrangement in monastrol treated cells. D.) Results from experiments in which cells were treated with monastrol cytospun onto a slide. Measurements were then performed by drawing an elipse around the area containing centromeres then the distance between the center of the elipse and either 8p11 loci or 8q24 loci with (eKT) and without (8q24) Ndc80 recruitment was measured. (*indicates p-value <0.01, ** p-value <0.05, Students t-test) SEM from triplicate experiments N = 54 cells. Diagram of chromosome 8 indicates position of FISH probes analyzed.

We have previously shown that the 8q24 locus is stably enriched in CENP-A and the inner kinetochore protein CENP-C. The latter is known to recruit outer kinetochore components [29]. Therefore, we attempted to dissect whether 8q24 forms an ectopic kinetochore, which could result in chromosomal instability. To test this, we investigated the localization of the outer most kinetochore protein responsible for binding microtubules, Ndc80, which is an accepted marker of kinetochores [27]. To enrich for cells in mitosis, SW480 cells were arrested with colcemid. We next performed IF/FISH for the 8q24 locus and Ndc80 (Fig 4B). In parallel, we also tested whether altering the amount of CENP-A at 8q24 might affect potential kinetochore recruitment. To this end, we knocked down the centromeric chaperone HJURP, which as we observe above, increases CENP-A levels at 8q24 (Fig 3 and Figures A and B in S5 Fig). After knockdown, we scored the percentage of cells with Ndc80 localized to 8q24 (Fig 4B and Figure A in S6 Fig). We observed that ~35% of cells transfected with a control siRNA exhibited modest colocalization of Ndc80 at 8q24. However, HJURP siRNA led to an increase, to ~51.3% colocalization of Ndc80 at 8q24. These data would support the hypothesis that ectopic kinetochore can weakly form at this locus, and that this colocalization is sensitive to perturbations in the balance between CENP-A and its chaperone.

We wanted to probe further whether these sites exhibited behavior consistent with ectopic kinetochores. We took advantage of an elegant assay that has been previously used to confirm spindle attachment of an artificially created ectopic kinetochore formed by targeted assembly of CENP-A at a LacO array [4]. In this experiment, cells were treated with the Eg5 inhibitor monastrol to inhibit separation of spindle poles in prometaphase. Consequently, cells arrest at a stage in which centromeres attached to this monopolar spindle are closer to the center of this structure, while telomeres are found at the periphery (Fig 4A–4C). Therefore, using the same assay, we tested whether 8q24 loci that co-stain with Ndc80 are more centrally located than sites without kinetochore components. We observed that 8q24 sites that were positive for Ndc80 (eKT) were on average 3.4 μm away from the center, compared to an average distance of 4.5 μm at sites that lacked Ndc80 staining (Fig 4D). In addition, we compared this to another locus on chromosome 8 that does not associate with CENP-A, and which is located at the 8p11 locus. Consistent with our measurements for 8q24 sites without Ndc80, we found that, on average, 8p11 was also located 4.5 μm from the center. Thus, presence of Ndc80 at 8q24 correlates with a 1.1 μm shift in the average position of this locus, suggesting that this locus might have become competent to form a weak kinetochore.

### Increased ectopic CENP-A leads to mitotic defects and DNA damage

**Fig 5:**
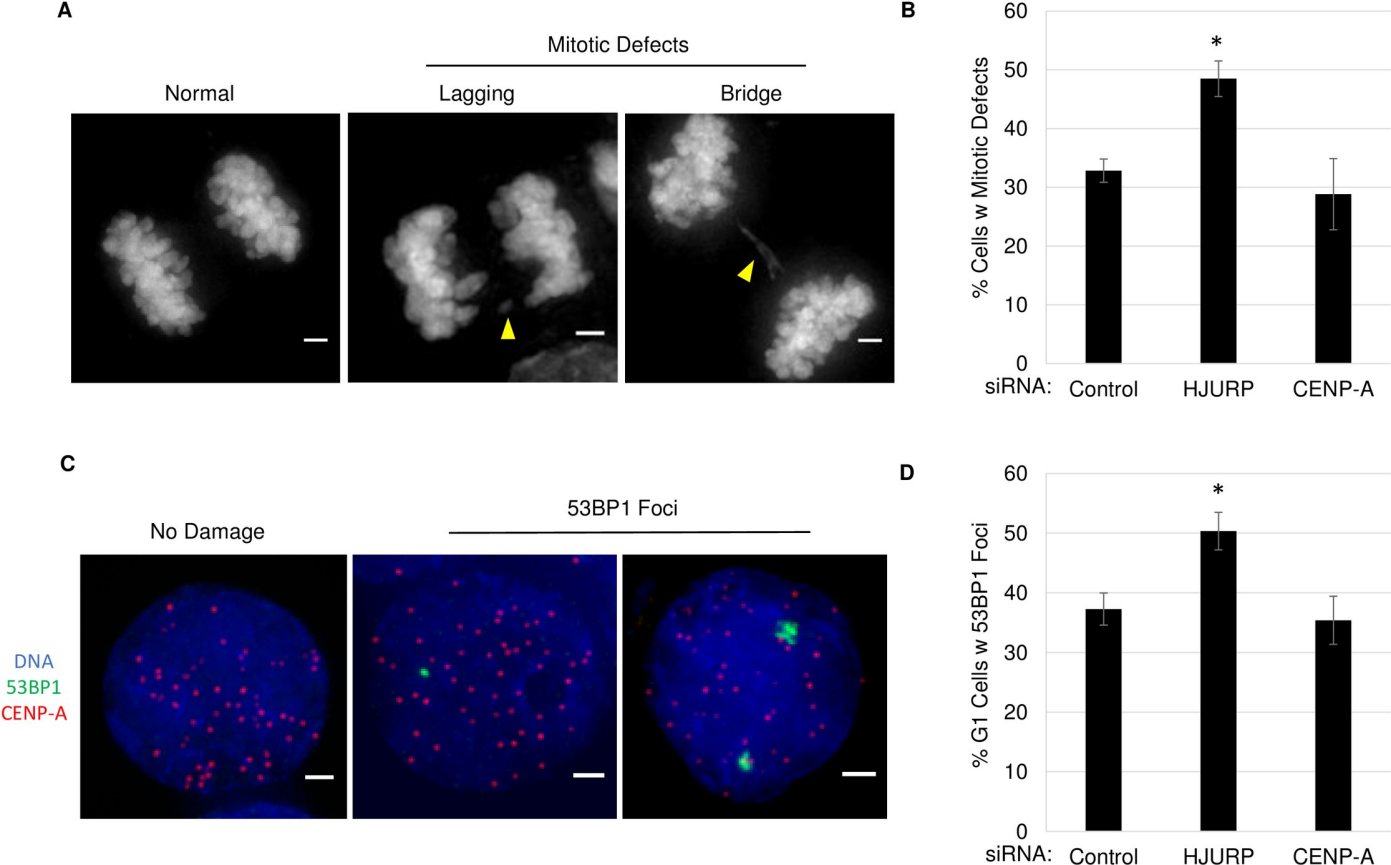
Increased ectopic CENP-A can lead to mitotic defects and DNA damage. A.) DAPI labeled DNA showing anaphase cells. Scale bar 1 μm. B.) Graph quantifying the number of cells with mitotic defects after siRNA. Error indicates SEM from 3 experiments n>65 cells per treatment. (*indicates P-value <0.01, Students t-test). C.) IF image showing G1 phase representative SW480 cells with or without DNA damage. IF for 53BP1, CENP-A and DAPI. Scale bar indicates 1 μm D.) Graph quantifying the percent of G1 phase cells with 53BP1 foci after siRNA. Error indicates SEM from 3 experiments n>250 per condition. (*indicates P-value <0.05, Students t-test).

We were also curious to test the effects of disrupting the balance between CENP-A and its chaperone. The results above suggest that even mild depletion of HJURP may lead to increased genomic instability, since ectopic CENP-A has been shown to result in mitotic defects [18]. A prediction then, is that increasing ectopic CENP-A, by knocking down the centromeric chaperone HJURP, might enhance mitotic defects. To test this, as before we performed an siRNA knockdown against HJURP for 72-hours and scored anaphase cells for mitotic defects including lagging chromosomes and chromosome bridges (Fig 5A and 5B). We observed that increasing ectopic CENP-A results in a 15% increase in cells with mitotic defects, from 32.8% to 48.5%, compared to control siRNA treated cells (p <0.01). We noted high levels of chromosomal instability (CIN) already present in these cells; however, our results show that driving mislocalization of CENP-A exacerbates the defects already present in this known CIN cell line [30]. It was plausible that this increase in mitotic defects could reflect a loss of CENP-A from endogenous centromeres. To test this, we also knocked down CENP-A itself for 72-hours and observed that both HJURP and CENP-A were depleted to a similar extent (Figure B in S2 Fig). Interestingly, we did not observe an appreciable increase in mitotic defects upon CENP-A knockdown. Since a similar reduction in CENP-A does not result in the same increase in mitotic defects, our data suggests that these mitotic defects are a result of an increase in ectopic CENP-A. However, given that the increase in CENP-A mislocalization is modest (~557 kb), we cannot rule out that these defects do not arise from indirect effects of knocking down HJURP. In addition, these findings indicate that large domains of CENP-A, such as those found at the centromere are not likely to be affected by short periods of CENP-A depletion. Thus, our results indicate that increasing ectopic CENP-A occurs much quicker than loss from pre-existing centromeres.

Mitotic defects such as lagging chromosomes and chromosome bridges have been associated with breakage-fusion-bridge cycles, in which a chromosome is pulled by both sides of the spindle resulting in a double strand break (DSB) following mitosis [31, 32]. Therefore, we next examined whether ectopic CENP-A correlates with increased DSBs in early G1. Once again, cells were treated with siRNA for HJURP or CENP-A, synchronized cells were then released into G1 phase, followed by IF for the DSB protein 53BP1 (Fig 5C and 5D). These experiments showed a significant increase in cells with DNA damage upon knockdown of HJURP, increasing from 37.2% to 50.3%. We saw no increase with knockdown of CENP-A alone, with 35.4% of cells positive for 53BP1. These data support the hypothesis that an appropriate balance between CENP-A and its chaperone is necessary to prevent genomic instability and DNA damage.

### Ectopic kinetochore recruitment correlates with amplification of the 8q24/*MYC* Locus and increased *MYC* expression

**Fig 6:**
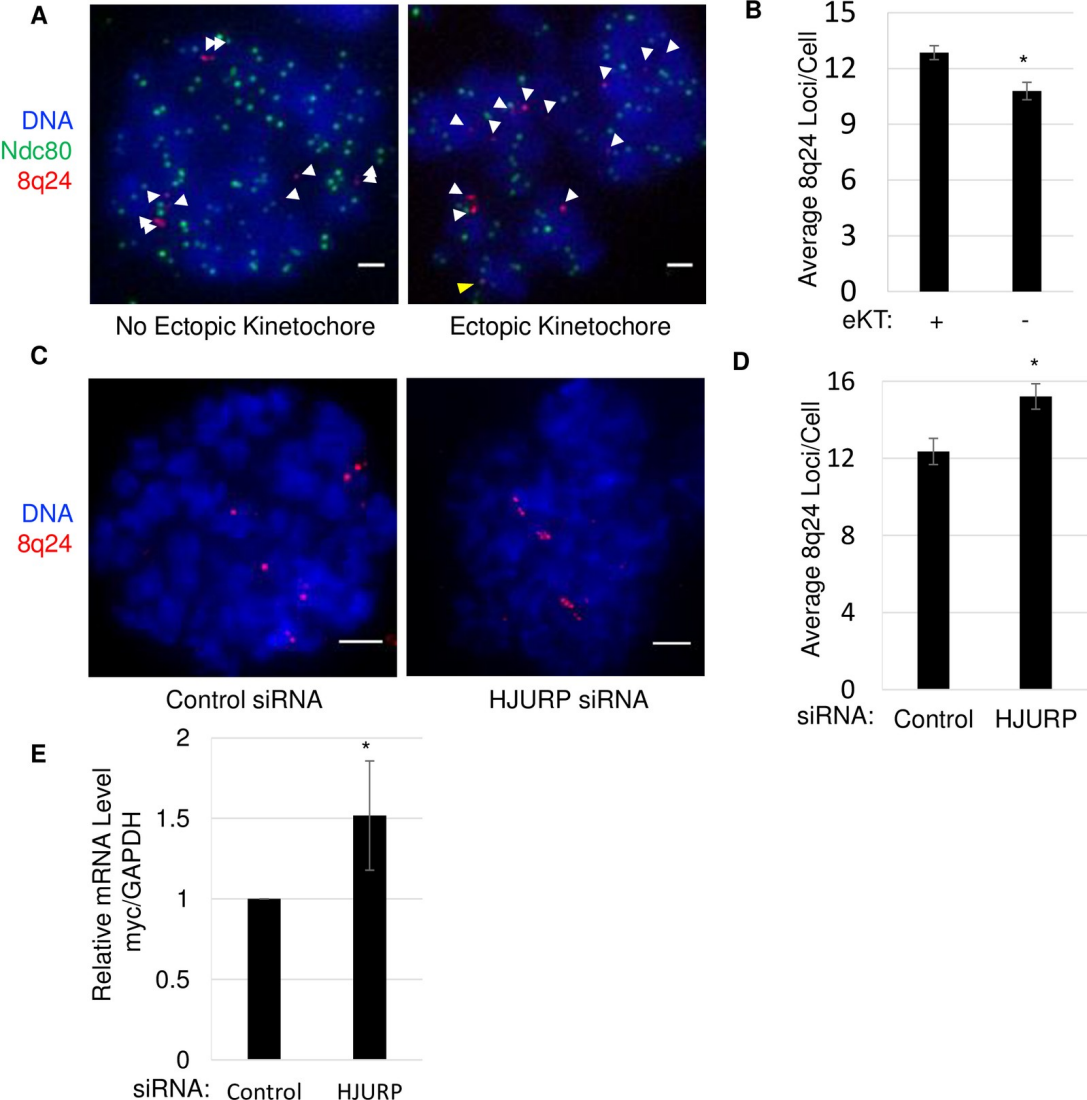
Ectopic kinetochore formation correlates with amplification of the 8q24/MYC locus and increased MYC expression. A.) Images showing IF in mitotic SW480 cells for Ndc80 and FISH for 8q24 locus. Scale bar indicates 1 μm. White arrows indicate FISH signals. Yellow arrowheads indicate colocalization. B.) Graph quantifying the number of 8q24 loci per cell for both ectopic kinetochore positive and negative cells, indicated by Ndc80 colocalization with at least one 8q24 FISH site. Error indicates SEM from 4 experiments n>200. (*indicates P-value <0.01, Students t-test). C.) Images showing DAPI stained DNA and FISH for the 8q24 locus in either control or HJURP knockdown cells. D.) Graph quantifying the number of 8q24 loci per cell for both control and HJURP knockdown cells. Error indicates SEM from 3 experiments n>190. (*indicates P-value <0.01, Students t-test). E.) Graph showing results of qPCR from mRNA harvested from either control or HJURP knockdown cells. Error measured from 3 experiments.

Previous studies examining human tumors with ectopic kinetochores observed the amplification of adjacent genes [33, 34]. Interestingly, the oncogene *MYC* has been found to be one of the most highly amplified genes in human tumors [35], including in SW480 cells, where we have observed robust amplification of this locus through sequencing and DNA FISH [17]. Therefore, we were interested to test whether cells with a potential ectopic kinetochore at 8q24 might display amplification of this region, compared to cells that do not. To accomplish this, we performed IF/FISH for Ndc80 at the 8q24 locus and then counted the number of 8q24 loci in mitotic cells (Fig 6A and 6B). In cells with no colocalization between Ndc80 and 8q24, there was an average of 10.8 8q24 loci per cell. In contrast, cells that displayed colocalization between Ndc80 and 8q24 had a slightly increased average of 12.9 8q24 loci per cell. These data suggest a mechanistic link between large domains of ectopic CENP-A and potential amplification of such domains.

A logical question that follows from these observations, is whether driving an increase in ectopic CENP-A would result in an increase in 8q24/*MYC* copy number. To ask this question, we treated cells with either control or HJURP siRNA and arrested cells in mitosis. The latter knockdown as discussed previously, increases ectopic CENP-A. Cells were then arrested in mitosis and FISH was performed to determine the number of 8q24 loci per cell (Fig 6C and 6D and Figure A in S7 Fig). Our results show a 22% increase in the number of loci per cell upon HJURP siRNA (p<0.01). In control siRNA treated cells, the average number of *MYC* loci was 12.4 per cell; in the HJURP knockdown cells, this number rose to 15.2 *MYC* loci per cell. However, when we examined another marker for chromosome 8, at the 8p11 locus, no increase in copy number was observed. Both, in the control and HJURP siRNA treated cells, 8p11 had an average number of 4.6 loci per cell. Taken together, these data suggest that *MYC* amplification at 8q24 is unlikely a result of aneuploidy of chromosome 8 (Figures A and B in S7 Fig).

*MYC* is a known oncogene and is highly expressed in a wide variety of cancer types [36]. Therefore, we were curious to test whether a modest increase in *MYC* copy number would result in quantifiable changes in *MYC* mRNA expression. To this end, we performed qRT-PCR and observed that *MYC* mRNA levels were increased 1.5-fold in HJURP siRNA treated versus control siRNA (Fig 6E). These data suggest that CENP-A mislocalization at 8q24, promotes amplification of nearby genes and leads to modest overexpression of *MYC*.

### The inner kinetochore protein CENP-C localizes to 8q24 in multiple tumor types

**Fig 7:**
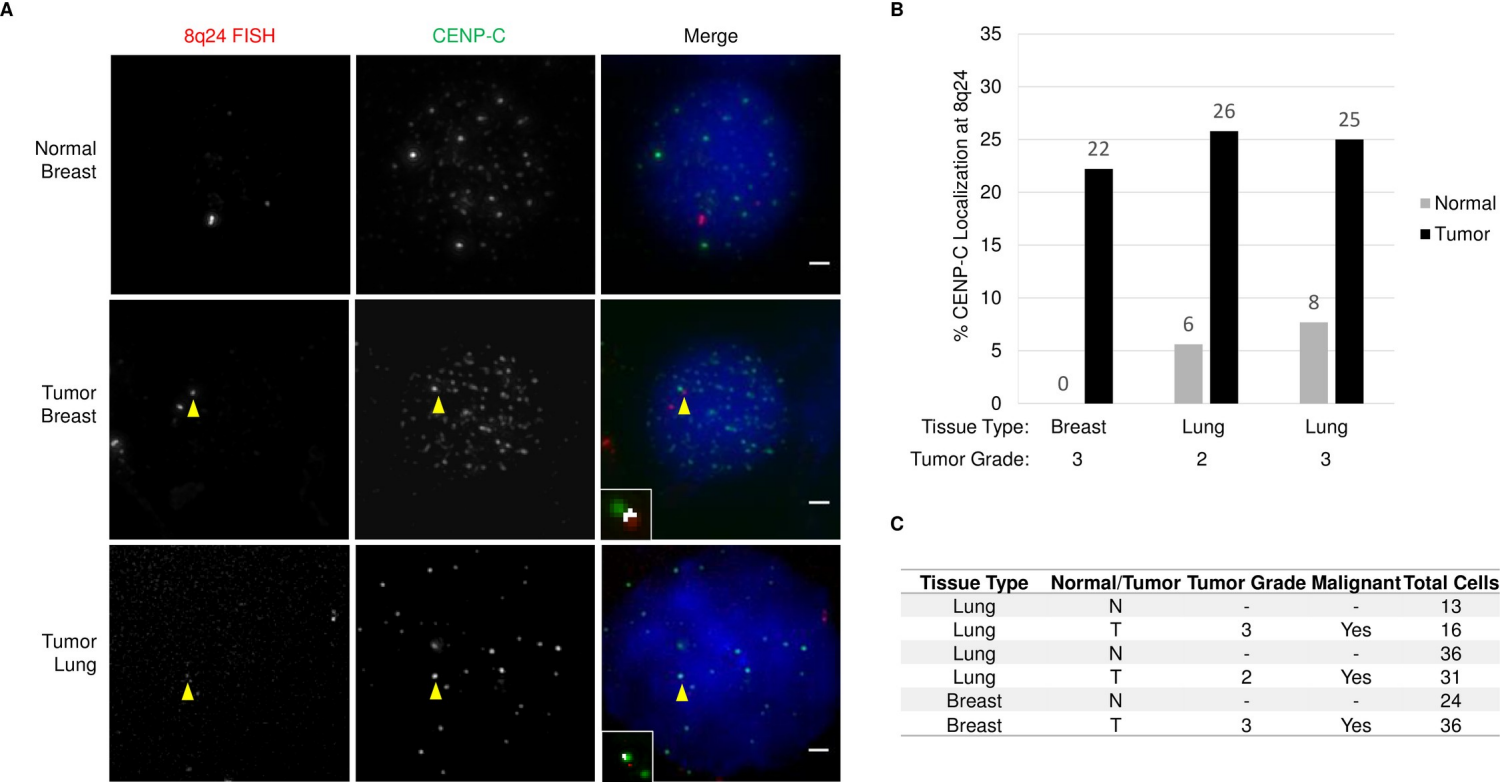
CENP-A localization to the 8q24 locus is common feature of cancer cells. A.) Images showing breast or lung cells isolated from normal or tumor tissue immunolabeled for CENP-C and FISH for 8q24. Yellow arrowheads indicate colocalization. Inset shows co-localization analysis performed using Image J; white is indicative of co-localization. Scale bar indicates 1 μm. B.) Graph quantifying the number of cells with colocalization between CENP-C and 8q24. C.) Table indicating the tissue type and tumor grade or each specimen examined.

Our previous work, and much of the work in this study was performed in the background of cells derived from colorectal tumors. We wanted to ask whether this phenomenon, of ectopic CENP-A occupying ectopic sites like 8q24, is specific to the tissue-of-origin, or is applicable across other tumor backgrounds. To this end, we obtained matched normal and tumor tissues from breast and lung biopsies, and scored for the presence of the CENP-A binding inner kinetochore protein CENP-C at 8q24. (Fig 7A–7C). We observed 0% colocalization in normal tissue, compared to 22% in a breast tumor sample from the same patient (Fig 7B). We also examined two grades of lung tumors to determine if there may be stage specific differences. In the normal tissue samples 5.6% and 7.7% of cells exhibited colocalization, however the grade 2 and grade 3 tumor samples both had ~25% colocalization. These data suggest that ectopic CENP-A occupancy of subtelomeric regions is applicable to diverse tumor types.

## Discussion

We have known for nearly 15 years that CENP-A is overexpressed in cancer cells [5–7]. The repercussions of increased levels and mislocalization of histone variants such as CENP-A are only now being uncovered. Here, we lend insights into this phenomenon, showing that when CENP-A is naturally overexpressed, it associates with the H3.3 chaperones HIRA and DAXX, resulting in ectopic assembly. Excitingly, we also observe that maintaining the balance between CENP-A and HJURP appears to be important to prevent mislocalization; when HJURP levels are low, pre-assembly CENP-A appears to be shunted into the H3.3 deposition pathway; conversely, when HJURP is overexpressed, this process is partially reversible [37]. Interestingly, although HJURP is frequently overexpressed in cancer, mutations in the HJURP gene have been identified which result in *decreased* levels of the protein and which are correlated with an increased risk of cancer [38–41]. As yet, it is unknown if those HJURP mutations result in CENP-A mislocalization. Our results in this report would suggest that mutations in HJURP which either decrease its amount relative to CENP-A, or *diminish its affinity* for CENP-A, can also lead to deleterious outcomes driven by ectopic localization of CENP-A. Moreover, our findings suggest that CENP-A is unique among histone variants because its chaperone choice is not dictated by a distinct structural feature, such as the G90 residue specific to H3.3 [42, 43]. Instead, CENP-A seems to bind promiscuously with H3.3 chaperones, but ordinarily relies on tight synchronization of expression of it and its chaperone HJURP during a small window in the cell cycle. Furthermore, post translational modifications have also been shown to influence the affinity of CENP-A for HJURP [44, 45]. These modifications, while not examined in this study, may play an important role in CENP-A localization, and are an exciting avenue for ongoing research.

Interestingly, we observe that modest imbalances between HJURP and CENP-A may have detrimental effects on genome stability. Our results suggest that this imbalance can result not just in ectopic assembly of CENP-A, but also in modest recruitment of other kinetochore components, which correlate with increased mitotic defects [46], and amplification of the underlying DNA. This latter phenomenon is widely observed in human tumors [33, 34]. Consistent with this idea, we present evidence that increasing CENP-A mislocalization and ectopic kinetochore formation may be one factor involved in the amplification and overexpression of *MYC*. An important question that arises from this work is whether ectopic CENP-A/CENP-C domains only transiently recruit outer kinetochore components, or whether can become neocentromeres. Indeed, future work will expand to test additional such ectopic loci for the presence and stability of several outer kinetochore components. It is also important to factor in complex genetic and epigenetic changes that are present in the context of any cancer. It is very likely that additional mutations and epigenetic changes, not exclusive to CENP-A mislocalization, will contribute to the observed genomic instability or MYC amplification. Thus, we think it will be important to determine the effects of CENP-A mislocalization in a variety of different contexts. Moreover, it will be of interest to investigate whether patient tumors that possess excess CENP-A and low HJURP levels also display *MYC* amplification.

This report presents additional insights into how CENP-A is mis-localized, and potential consequences of its invasion at ectopic sites. An important future aim will be to address an intriguing question: are there underlying genetic or epigenetic features which make subtelomeric sites attractive spots for CENP-A localization? One possibility is linked to previous work which suggested that overexpressed CENP-A is especially stable at heterochromatin boundaries, suggesting that subtelomeric sites may be ideal [27]. Precisely how ectopic kinetochores are maintained in human cells has not been elucidated at the mechanistic level. Our data suggests an unanticipated role for H3.3 chaperones, and for processes that promote nucleosome turnover, in the maintenance of such ectopic domains. Consequently, these findings present a novel direction of research which should prove very interesting to pursue in the context of neocentromere establishment and maintenance.

In sum, in this report we provide evidence for a “chaperone-balance” model of CENP-A mislocalization [22], involving both chaperones in the H3.3 pathway. We also shed light on the consequences of this mis-regulation, including chromosomal instability and amplification of the 8q24/*MYC* locus. Future work in this area will be important to increase our understanding of the changes in the epigenetic landscape that occur in cancer and therefore allow us to predict and prevent their occurrence.

## Materials and methods

### siRNA

siRNAs were transfected using electroporation Lonza Kit V, program L-024 into 1x10^6^ SW480 cells. siRNAs for HIRA and DAXX as described in [16], CENP-A and HJURP as described in [47]. Sequences listed in (S1 Table).

### Combined immunofluorescence and *fluorescent in situ hybridization*

Preparation of matched tumor and normal tissue, labeling of BAC probe and combined IF/FISH performed as in [17]. Analysis of colocalization in these experiments was assayed by looking at individual z-sections from z-stacks.

### Cell synchronization and immunofluorescence

SW480 cells were transfected with siRNAs for 72-hours total. Before harvesting the cells were treated with the CDK inhibitor RO-3306 for 16 hours to arrest cells in G2 phase [48]. The cells were released from G2 by washing the cells 3 times with warm media. Anaphase cells were obtained after a 1.5 hour release then treated with DAPI to analyze mitotic defects. For G1 phase, cells were released for 2 hours then immunofluorescence was performed as in [49]. Mitotic cells were obtained by treating cells with 100 ng/ml colcemid for 6–8 hours. Cell synchronization by double thymidine block performed as in [17].

Monastrol treatment performed as in [4].

### Western blots and antibodies

For western blots Invitrogen novex 4–20% gels (cat# XP04200) were used and transfers were performed using transblot turbo transfer packs (cat# 1704158). Blocking was performed using licor PBS blocking buffer (cat#927–40000).

CENP-A Abcam ab13939; HJURP Sigma Aldrich (HPA008436); DAXX Santa Cruz Biotech M-112; HIRA EMD Millipore WC119; Tubulin Santa Cruz Biotech DM1A; CENP-C MBL; 53BP1 Santa Cruz Biotech H-300; Ndc80 GeneTex 9G3.23; CENP-A IP: ACA Serum (Centromere Ab Positive Serum) BBI Solutions.

### Immunoprecipitation

The soluble fraction of CENP-A was obtained by suspending cells in PBS with 0.5% NP-40 for 5 minutes on ice. Nuclei were then pelleted via centrifugation for 1 minute at 1000 RPM. Nuclei were once again resuspended in PBS NP-40 buffer and pelleted via centrifugation as before. Supernatants were pre-cleared via centrifugation at 12000 RPM for 10 minutes and the resulting supernatant was used in the IP.

### ChIP-Seq library construction and sequencing

Native ChIP was performed as in [50] with the exception of a longer MNase digest of 15 minutes. For each of four independent replicates (or duplicates for double knockdowns), libraries were constructed using the Illumina TruSeq ChIP Sample Prep Kit (#IP-202-1012-1024). In each replicate, ChIP libraries were sequenced on NextSeq flowcells using V2 chemistry (Illumina, Inc., San Diego, CA). Sequencing was performed for 76 cycles, in either single- or paired-end mode. Sequencing depths per replicate ranged from 95 million to 315 million reads. (S2 Table) summarizes samples and read depths.

### ChIP-Seq read mapping and peak calling

Reads were demultiplexed with the bacl2fastq2 program (v2.17.1.4, Illumina, San Diego, USA). Raw reads that passed Illumina quality filters were trimmed for adapter sequence and low quality using Trimmomatic [51]. These processed reads were aligned to the hg19 assembly with Bowtie2 v2.2.6 [52] with default alignment parameters, reporting a single best alignment where there are multiple alignments possible. To reduce spurious peak calling in repetitive sequence, we filtered these alignments for alignment quality (which incorporates uniqueness) using samtools [53] with a quality criterion of ten or greater. Depth normalized browser tracks were generated from these data using Deeptools [54]. Raw data have been deposited under GEO accession GSE120230.

Peaks were called using the MACS peak caller [55], using default parameters, comparing each ChIP sample to its respective input as control. Coordinate intersections with bedtools [56] were used to define regions concordant between replicates.

### ChIP-Seq analysis

We obtained coordinates for centromere boundaries for the hg19 assembly from the UCSC genome browser [57]. We defined peaks as ectopic when they fell completely outside these centromere boundaries. To create a set of robustly replicated peaks within each sample, peaks for each replicate were merged with Bedtools to create a single reference peak set for each sample. These reference peaks were then considered replicated if they overlapped peaks called in least three replicates separately (for schematic, see Fig 1A). Comparative results between samples are presented for the replicated peaks.

To compare enrichment between samples, we summed the bases within peaks, by subtracting start and end coordinates (“Peak coverage,” Fig 1). Base-level peak intersections were performed using Bedtools, parsed with an in-house script and plotted in R with the VennDiagram package (see Fig 1C for schematic).

To confirm that technical differences (eg sequencing depth, single/paired ends, replicate number) were not responsible for ChIP-Seq peak coverage changes, we removed technical differences by random sampling. First-of-mate reads were extracted from paired-end samples, and the two highest-depth replicates were pooled together for each sample. From these pools, three random samples of equal depth (75 million reads) were extracted for each IP. One random sample of 100 million reads was obtained for each input sample. Peak calling was then performed with these subsets. Mean and standard deviation of peak coverage was calculated across trials for each experiment. All computation was performed in the R statistical programming environment (https://www.r-project.org/).

## Supporting information

S1 Fig A.) Flow cytometry analysis of SW480 cells synchronized using a double thymidine block then released for the indicated time points. B.) RT-PCR showing gene expression of the indicated gene. C.) Graph showing expression levels at the indicated time-points.
(TIF)

S2 Fig A.) Western blots showing knockdown of the indicated protein 72-hours after transfection with siRNA. HIRA levels were too low for whole cell lysates, so nuclear extracts were prepared. B.) Quantitation of western blots measuring the depletion of either HJURP or CENP-A. SEM from triplicate experiments. C.) Western blots showing knockdown of CENP-A and HJURP 72-hours after transfection of siRNA.
(TIF)

S3 Fig A. Bar chart showing the mean peak coverage, in kilobases, of ectopic CENP-A peaks from 3 random samplings of reads from pooled ChIP-seq experiments. Standard deviations are shown in error bars. Starred comparisons show p<0.01, t-test.
(TIF)

S4 Fig A.) A.) Western blots showing the results of an IP experiment in which GFP or GFP-CENP-A was IP’d from stable cell lines. B.) Graph showing the relative expression levels of each chaperone in HeLa cell line compared to SW480 colon cancer cells. Results are representative of triplicate experiments. C.) Western blot showing levels of each chaperone in the indicated cell line. D.) Total protein staining used to normalize the chaperone levels in A.
(TIF)

S5 Fig A.) Browser shots from CENP-A ChIP-seq in either control or HJURP treated SW480 cells. B.) Fold change in replicated peaks in the 8q24 region in cells treated with the indicated siRNA. C.) Bar chart showing the mean peak coverage, in kilobases, of ectopic CENP-A peaks from 3 random samplings of reads from pooled ChIP-seq experiments. Standard deviations are shown in error bars. Starred comparisons show p<0.05, t-test. D.) Western blots showing expression of GFP tagged proteins in stable cell lines used for in CENP-A ChIP-seq overexpression experiments. Arrowhead indicates GFP-HJURP protein and the asterisk marks a background band directly below it.
(TIF)

S6 Fig A.) Image showing monastrol treated cell. FISH for the 8q24 locus and IF for the Ndc80 protein was performed on SW480 cells. DAPI in blue. Yellow arrowheads indicate colocalization. Inset shows automated co-localization analysis performed using Image J; white is indicative of co-localization. Scale bar indicates 1 μm.
(TIF)

S7 Fig A.) Images showing FISH for 8q24 in cells treated with either control or HJURP siRNA for 72-hours then arrested in mitosis. B.) Images showing FISH for 8P11 in cells treated with HJURP siRNA for 72-hours then arrested in mitosis. C.) Graph showing average number of 8p11 loci in control or HJURP treated cells after 72-hours.
(TIF)

S1 Table siRNA sequences used in the chaperone knockdown experiments. (XLSX)

S2 Table ChIP-Seq samples and read depths. (XLSX)
