# Correction: HJURP antagonizes CENP-A mislocalization driven by the H3.3 chaperones HIRA and DAXX

**DOI:** 10.1371/journal.pone.0207631

**Published:** 2018-11-12

**Authors:** The PLOS ONE Staff

## Notice of republication

Due to an error introduced during the typesetting process, incorrect versions of Figs 1–7, and Supporting Information files S1-S7 Fig and S1-S2 Table were published in error. The publisher apologizes for these errors. This article was republished on November 02, 2018 to correct for these errors. Please download this article again to view the correct version.
