# Supplementary figures and images for: HJURP antagonizes CENP-A mislocalization driven by the H3.3 chaperones HIRA and DAXX
Source: PLoS One. 2018 Oct 26;13(10):e0205948. doi: 10.1371/journal.pone.0205948 (PMC6203356; doi:10.1371/journal.pone.0205948)

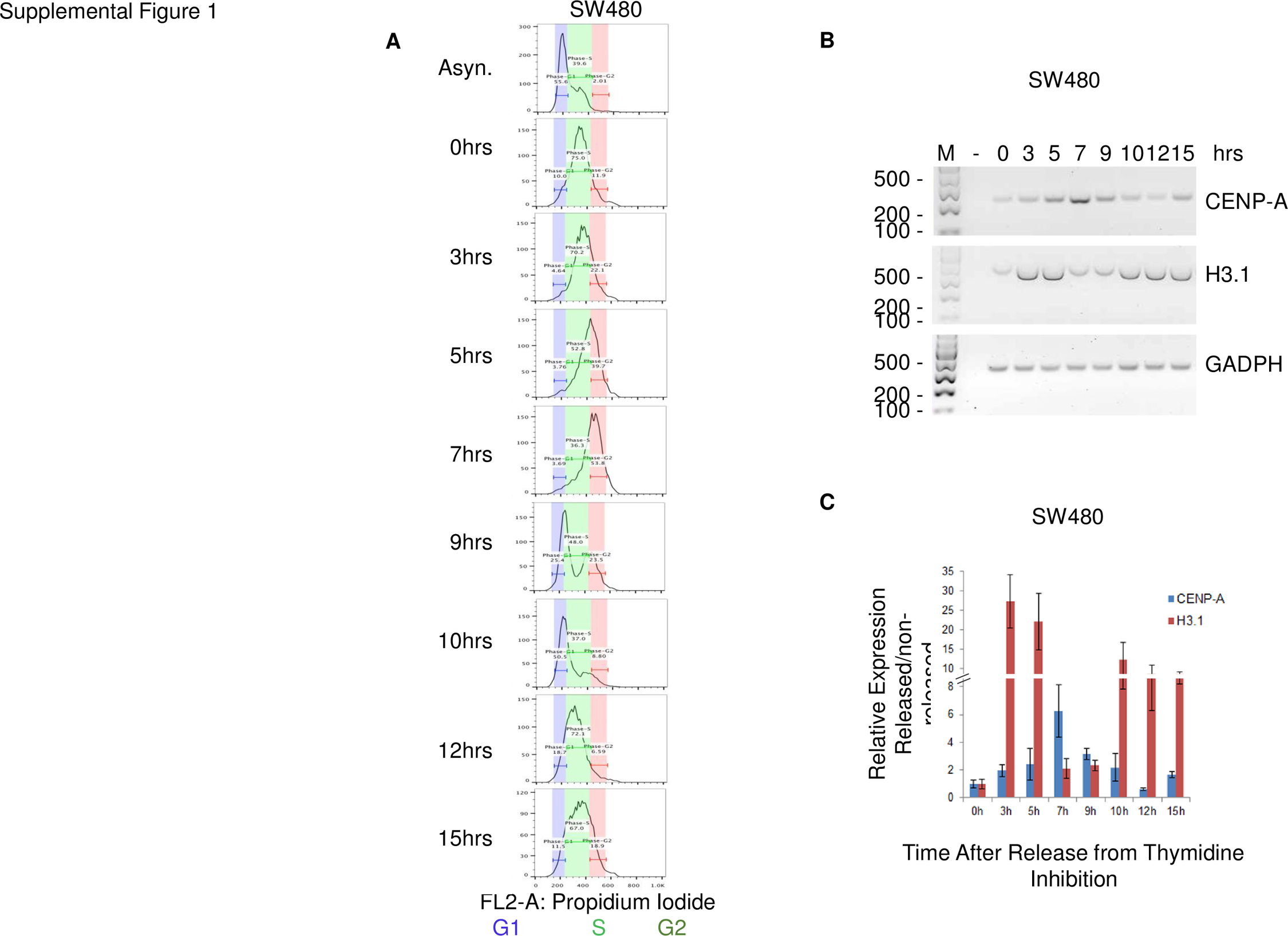

Supplement: S1 Fig — A.) Flow cytometry analysis of SW480 cells synchronized using a double thymidine block then released for the indicated time points. B.) RT-PCR showing gene expression of the indicated gene. C.) Graph showing expression levels at the indicated time-points. (TIF) [file pone.0205948.s001.tif]

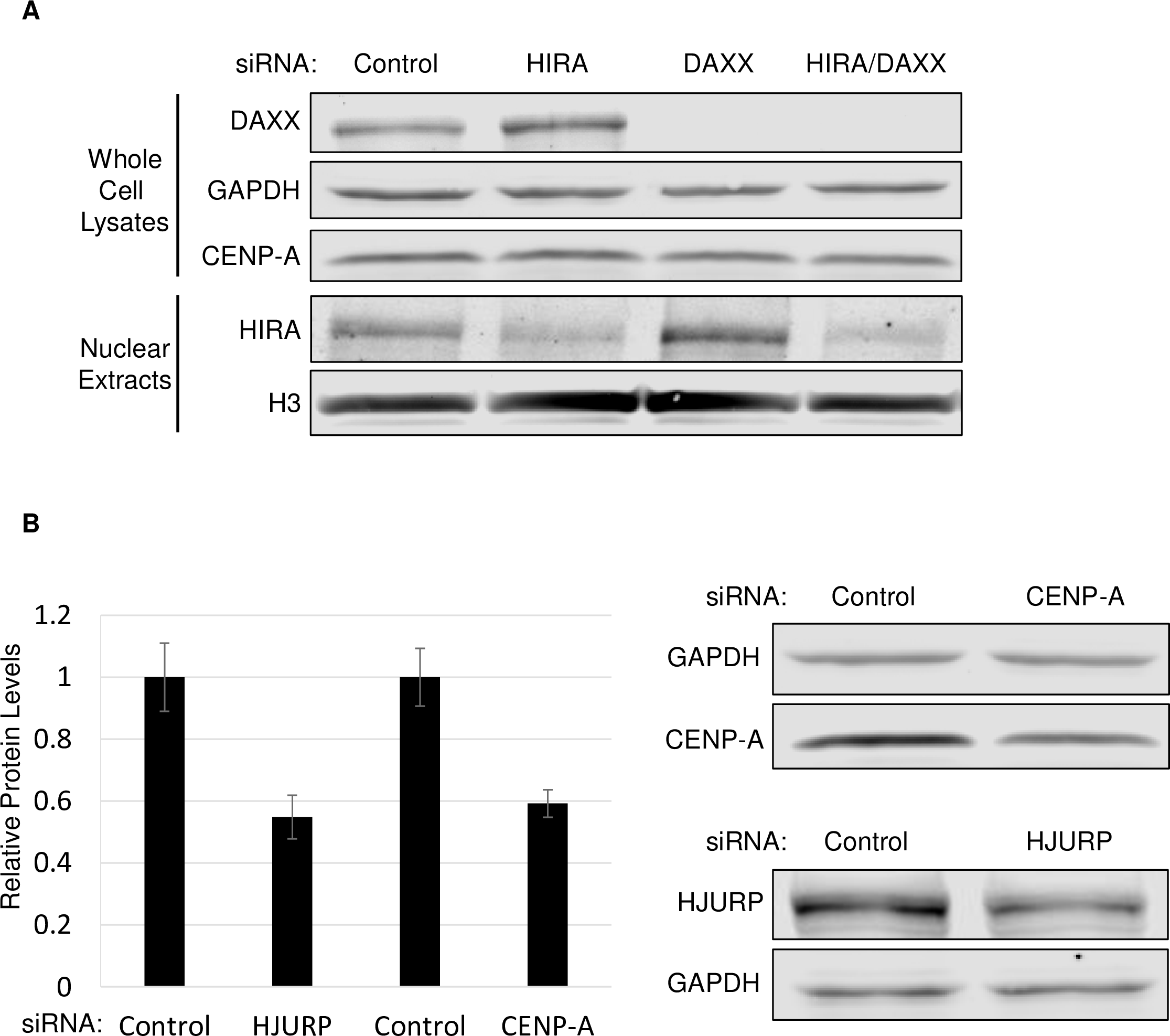

Supplement: S2 Fig — A.) Western blots showing knockdown of the indicated protein 72-hours after transfection with siRNA. HIRA levels were too low for whole cell lysates, so nuclear extracts were prepared. B.) Quantitation of western blots measuring the depletion of either HJURP or CENP-A. SEM from triplicate experiments. C.) Western blots showing knockdown of CENP-A and HJURP 72-hours after transfection of siRNA. (TIF) [file pone.0205948.s002.tif]

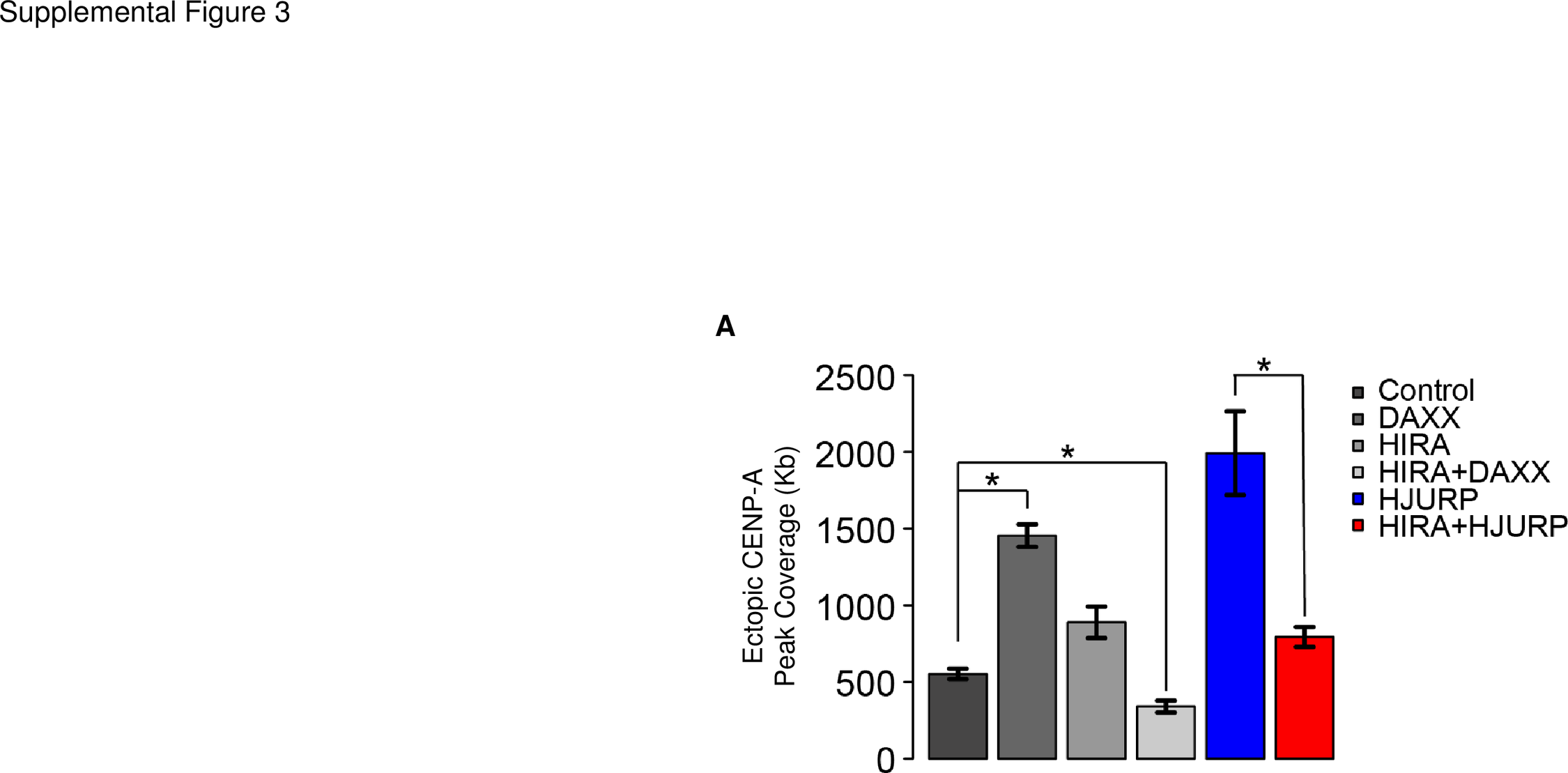

Supplement: S3 Fig — A. Bar chart showing the mean peak coverage, in kilobases, of ectopic CENP-A peaks from 3 random samplings of reads from pooled ChIP-seq experiments. Standard deviations are shown in error bars. Starred comparisons show p<0.01, t-test. (TIF) [file pone.0205948.s003.tif]

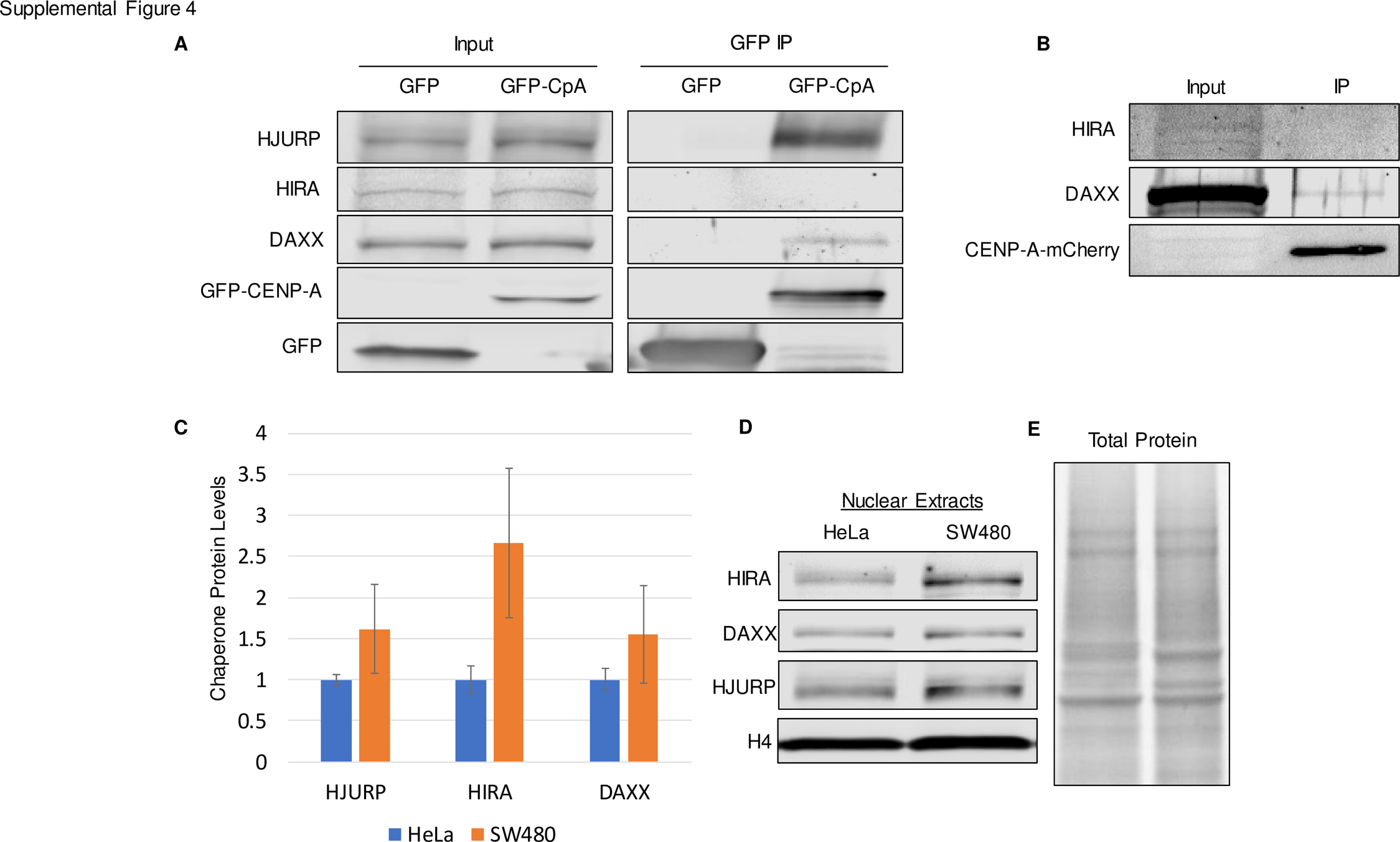

Supplement: S4 Fig — A.) A.) Western blots showing the results of an IP experiment in which GFP or GFP-CENP-A was IP’d from stable cell lines. B.) Graph showing the relative expression levels of each chaperone in HeLa cell line compared to SW480 colon cancer cells. Results are representative of triplicate experiments. C.) Western blot showing levels of each chaperone in the indicated cell line. D.) Total protein staining used to normalize the chaperone levels in A. (TIF) [file pone.0205948.s004.tif]

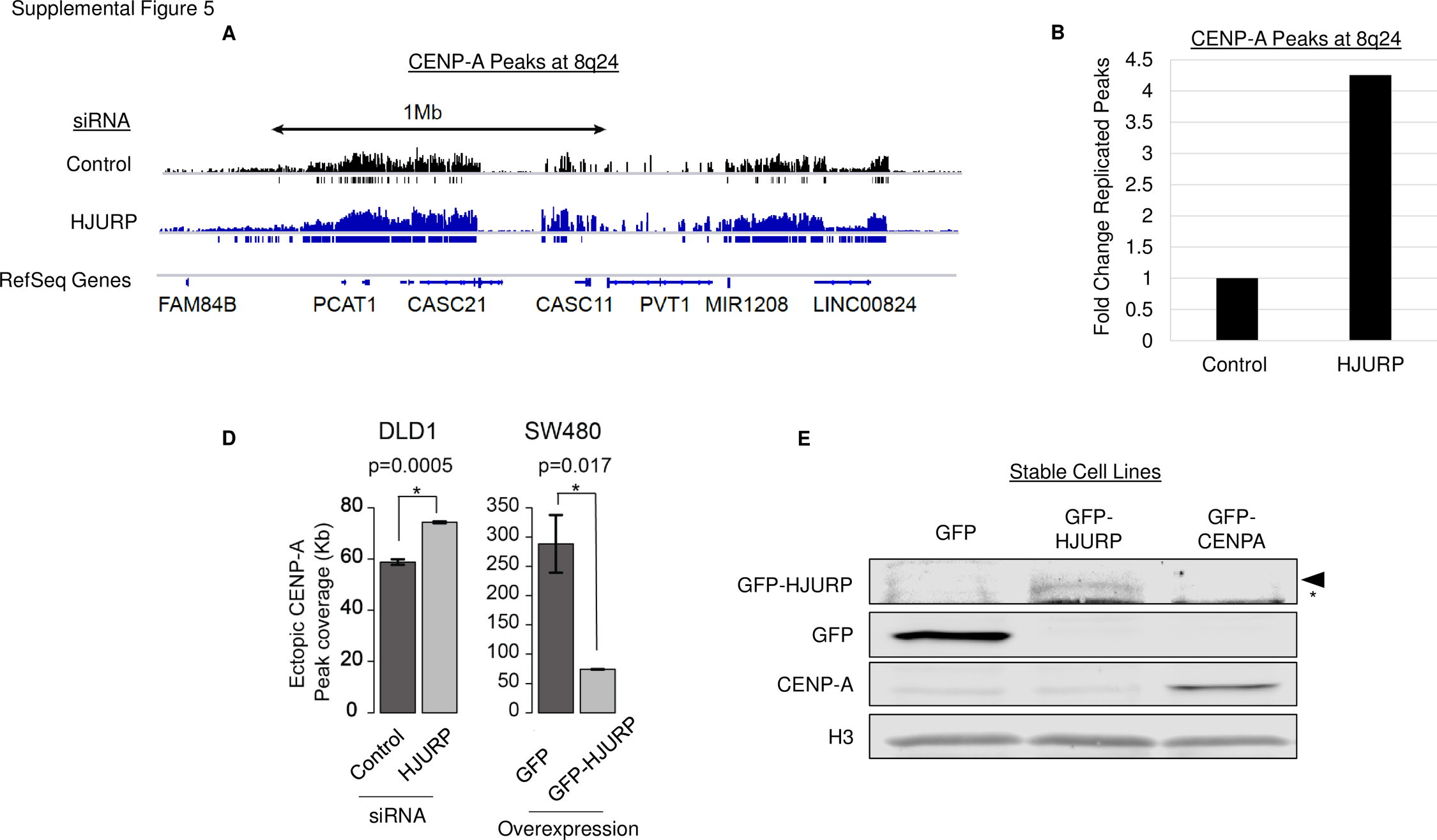

Supplement: S5 Fig — A.) Browser shots from CENP-A ChIP-seq in either control or HJURP treated SW480 cells. B.) Fold change in replicated peaks in the 8q24 region in cells treated with the indicated siRNA. C.) Bar chart showing the mean peak coverage, in kilobases, of ectopic CENP-A peaks from 3 random samplings of reads from pooled ChIP-seq experiments. Standard deviations are shown in error bars. Starred comparisons show p<0.05, t-test. D.) Western blots showing expression of GFP tagged proteins in stable cell lines used for in CENP-A ChIP-seq overexpression experiments. Arrowhead indicates GFP-HJURP protein and the asterisk marks a background band directly below it. (TIF) [file pone.0205948.s005.tif]

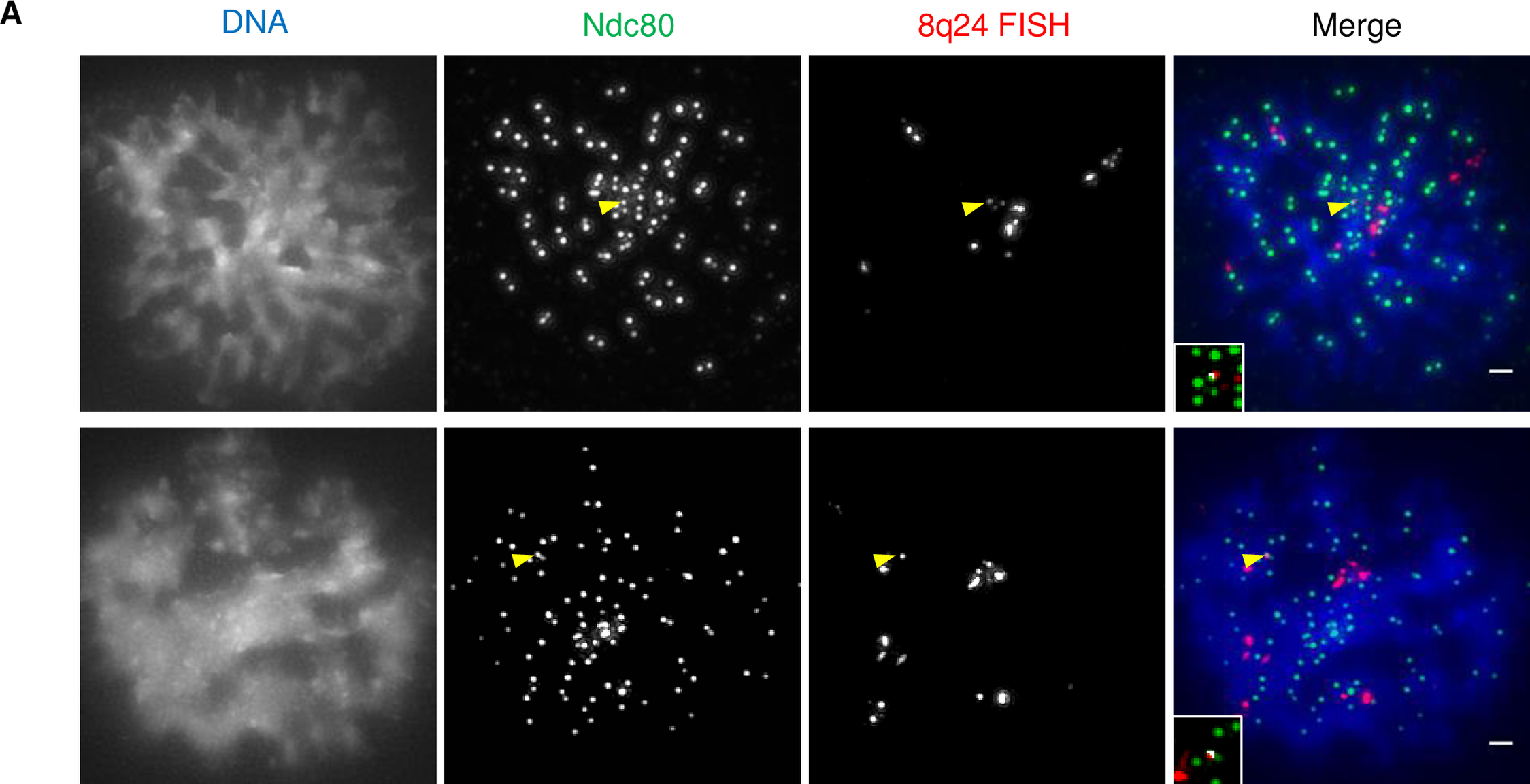

Supplement: S6 Fig — A.) Image showing monastrol treated cell. FISH for the 8q24 locus and IF for the Ndc80 protein was performed on SW480 cells. DAPI in blue. Yellow arrowheads indicate colocalization. Inset shows automated co-localization analysis performed using Image J; white is indicative of co-localization. Scale bar indicates 1 μm. (TIF) [file pone.0205948.s006.tif]

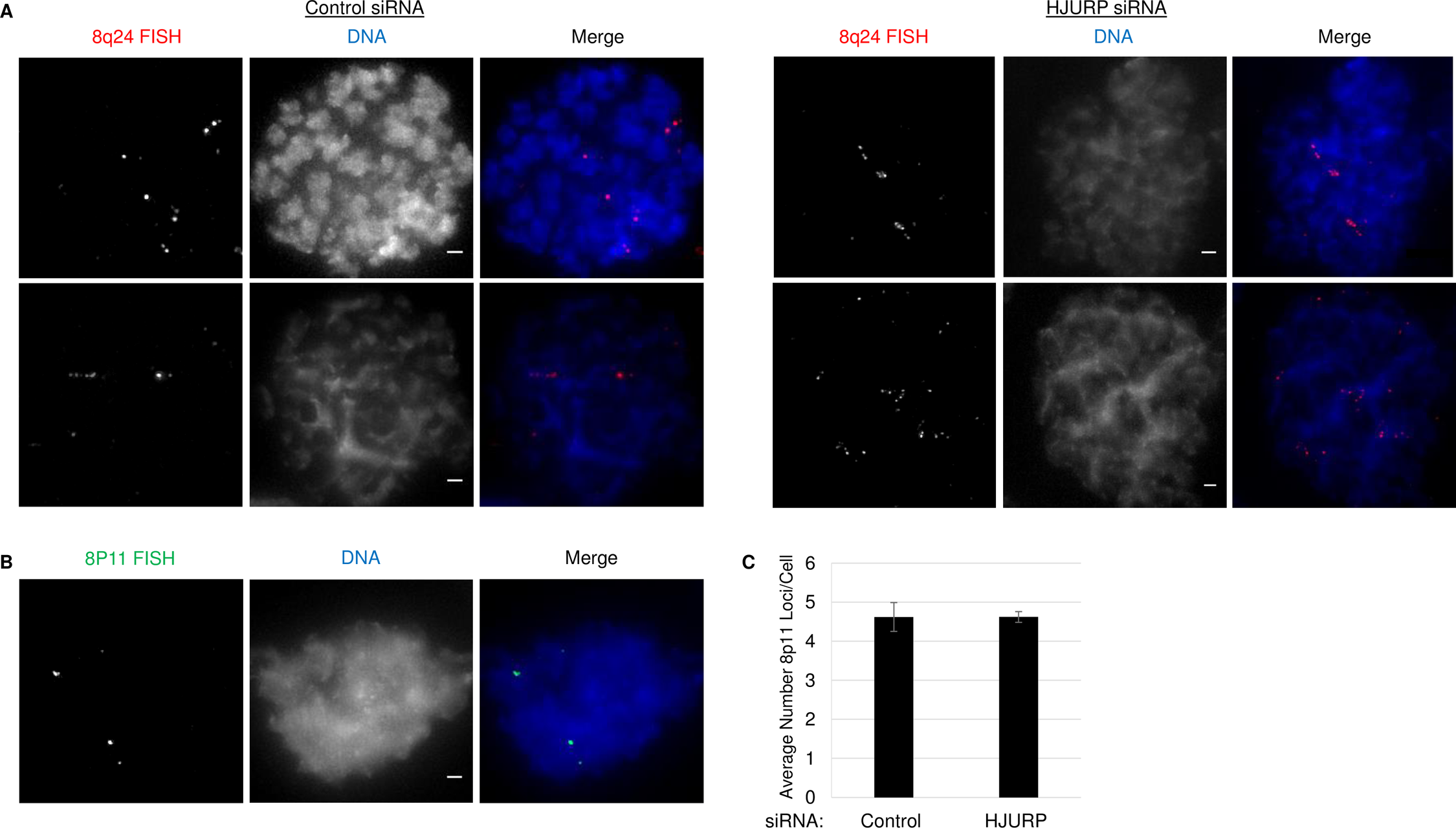

Supplement: S7 Fig — A.) Images showing FISH for 8q24 in cells treated with either control or HJURP siRNA for 72-hours then arrested in mitosis. B.) Images showing FISH for 8P11 in cells treated with HJURP siRNA for 72-hours then arrested in mitosis. C.) Graph showing average number of 8p11 loci in control or HJURP treated cells after 72-hours. (TIF) [file pone.0205948.s007.tif]
